# Predictor Role of *VKORC1* rs9923231, *CYP4F2* rs2108622, and *GGCX* rs11676382 Polymorphisms of 5 Years Mortality of Patients with Acute Ischemic Stroke

**DOI:** 10.3390/medicina61101760

**Published:** 2025-09-28

**Authors:** Silvina Iluţ, Valer Donca, Antonia Eugenia Macarie, Ştefan Cristian Vesa, Raluca Maria Pop, Vitalie Văcăraş, Diana Şipoş-Lascu, Ioana Cristina Bârsan, Lăcrămioara Perju-Dumbravă, Ovidiu Sorin Chiroban, Camelia Alexandra Coadă, Anca Dana Buzoianu

**Affiliations:** 1 2nd Department, Faculty of Medical Assistance and Health Sciences, “Iuliu Hațieganu” University of Medicine and Pharmacy, 400012 Cluj-Napoca, Romania; silvina.ilut@umfcluj.ro; 2 Department of Geriatrics-Gerontology, “Iuliu Haţieganu” University of Medicine and Pharmacy, 400012 Cluj-Napoca, Romania; macarieantonia@yahoo.com; 3 Department of Pharmacology, Toxicology and Clinical Pharmacology, “Iuliu Haţieganu” University of Medicine and Pharmacy, 400337 Cluj-Napoca, Romaniaraluca.pop@umfcluj.ro (R.M.P.); abuzoianu@umfcluj.ro (A.D.B.); 4 Department of Neurosciences, Faculty of Medicine, “Iuliu Hațieganu” University of Medicine and Pharmacy, 400012 Cluj-Napoca, Romania; vvacaras@umfcluj.ro (V.V.); dia_lascu@yahoo.com (D.Ş.-L.); cristinabarsan13@gmail.com (I.C.B.); lperjud@umfcluj.ro (L.P.-D.); 5 Department of Forensic Medicine, Faculty of Medicine, “Iuliu Hațieganu” University of Medicine and Pharmacy, 400012 Cluj-Napoca, Romania; chiroban.ovidiu@umfcluj.ro; 6 Department of Morpho-functional Sciences, “Iuliu Haţieganu” University of Medicine and Pharmacy, 400012 Cluj-Napoca, Romania; coada_camelia_alexandra@elearn.umfcluj.ro

**Keywords:** acute ischemic stroke, VKORC1, mortality, predictor

## Abstract

*Background and Objectives*: The aim of this study was to evaluate the potential predictive value of *VKORC1*, *CYP4F2*, and *GGCX* polymorphisms, as well as other clinical and demographic factors, for 5-year mortality in patients with acute ischemic stroke (AIS). *Materials and Methods*: The study enrolled 252 patients who were consecutively hospitalized for AIS. Demographic data, comorbidities, and laboratory tests were collected. Genotyping of the *VKORC1* rs9923231 (-1639G > A; VKORC1*2), *CYP4F2* rs2108622 (1347C > T), and *GGCX* rs11676382 (12970C > G) polymorphisms was performed. Mortality was noted if it occurred within five years following the 30 days after discharge, using the National Health Insurance House registry. *Results*: Death was recorded in 71 (28.1%) patients. In multivariate analysis the following variables were independent variables associated with 5-year mortality: age > 72 years (OR 2.83 (95%CI 1.32; 6.08), *p* = 0.007), a lesion volume > 12.6 mL (OR 4.05 (95%CI 2.05; 7.99), *p* < 0.001), and an NIHSS score > 7 (OR—2.64 (95%CI 1.31; 5.31), *p* = 0.006). *VKORC1* (-1639G > A) SNP m/m variant was only marginally associated with mortality. *Conclusions*: In this study which included AIS patients, *VKORC1*, *CYP4F2*, and *GGCX* polymorphisms did not independently predict mortality. The *VKORC1* variant was only marginally associated with mortality, but this was attenuated after correction for multiple testing. Advanced age, NIHSS score, and the lesion volume were independent predictors of long-term mortality in AIS patients.

## 1. Introduction

Acute ischemic stroke (AIS) is one of the leading causes of death in patients with cardiovascular diseases (1 in 6 deaths from cardiovascular disease) [1]. It is also a major source of short- and long-term disability, which comes with a high socio-economic cost [2]. Despite improvements in acute management (intravenous thrombolysis within 4.5 h and endovascular thrombectomy for anterior-circulation large-vessel occlusion within 6 h), the long-term outcome for AIS patients is still relatively poor [1]. Studies showed that these interventions more likely improve the short/medium term outcomes rather than long-term survival [3].

In Romania, AIS is a major public health issue, with an incidence that rose from 87.6 to 201 per 100,000 between 2007 and 2015, which indicates a worse-than-average burden compared to Western Europe [4]. Despite some improvements in stroke care in recent years (an increase in the thrombolysis rate from 0.8% to 5.4% between 2017 and 2022), mortality and long-term disability rates remain among the highest in the European Union. The 5-year mortality rate after an ischemic stroke in Romania is estimated at 21.6%, almost twice the European average [5].

Current research is increasingly focused on identifying genetic and molecular markers that could help more accurately predict the long-term mortality risk in AIS. To date, no studies have investigated the potential predictive role of polymorphisms involved in vitamin K (VK) metabolism for long-term mortality in AIS patients who are not receiving treatment with VK antagonists. Studies on European populations showed that the *VKORC1* rs9923231 A-allele is common (minor-allele frequency ~40–45%); in Romanians, an A-allele frequency as high as 42% was reported, comparable to other European cohorts [6,7]. In European-Americans, the VKORC1 haplotype Group A associated with a low dose of warfarin has a prevalence of ~35%, and *VKORC1* variants explain a larger share of dose variability than in African-Americans [8]. The *CYP4F2* rs2108622 minor allele is frequent in Europeans, European Americans, and Central/Eastern European populations (25–30%; 30%; 23–32%, respectively) [9,10,11]. The *GGCX* rs11676382 minor allele is much less frequent than *VKORC1* or *CYP4F2* in Europeans (7–12%) [12]. We previously investigated the implication of *CYP4F2* and *VKORC1* polymorphisms in carotid plaque formation and risk of AIS, and we showed that polymorphisms in the *VKORC1* and *CYP4F2* genes might increase the probability of plaque carotid formation and may increase the risk of AIS in patients without a determined embolic source [13,14]. Several of these genetic variants involved in VK metabolism may influence long-term outcomes after AIS. The *VKORC1* rs9923231 (-1639 G > A) polymorphism reduces VKORC1 expression, which will lead to a reduced recycling of vitamin K and will decrease the activation of VK-dependent proteins [15]. These proteins, mainly matrix Gla protein (MGP) and growth arrest-specific gene 6 (Gas6), have a very important role in arterial integrity, inhibition of calcification, and neurovascular repair [16]. The *GGCX* rs11676382 polymorphism can alter the γ-carboxylation process which is required for functional activation of these proteins. This can further increase the arterial dysfunction [17]. The *CYP4F2* rs2108622 (V433M) variant reduces hepatic catabolism of vitamin K1, which can decrease its systemic availability and affect the arterial homeostasis [18]. Because VK-dependent proteins maintain vascular integrity, perturbations in the VKORC1–GGCX pathway (and VK catabolism via CYP4F2) may influence stroke outcomes—even in the absence of VK antagonists.

Clinical, demographic, imaging, and biochemical data are used alone or in combination as predictors for long-term mortality of patients with AIS. Among them, advanced age, male gender, AIS severity (quantified by NIH Stroke Scale (NIHSS)), comorbidities (atrial fibrillation (AF), diabetes mellitus (DM)), and neuroimaging markers (lesion volume, white matter damage) were proved to be associated with an increased risk of death [19,20,21,22]. Despite using these multiple and diverse factors, current prognostic models lack precision and misidentify biologically vulnerable patients with apparently similar clinical/biochemical/imaging profiles.

Taking into consideration the AIS burden and the role of VK in vascular diseases, we evaluated the prognostic value of *VKORC1* rs9923231, *CYP4F2* rs2108622, and *GGCX* rs11676382 polymorphisms for 5-year mortality after AIS, together with established clinical and imaging predictors (age, NIHSS, lesion volume, comorbidities), in patients not treated with VK antagonists.

## 2. Materials and Methods

This was a prospective, longitudinal, analytical cohort study. Patients diagnosed with AIS admitted at the Department of Neurology of the Emergency County Hospital of Cluj-Napoca between March 2019 and February 2020 were enrolled in the study. The study protocol was approved by the Ethics Committee of “Iuliu Hațieganu” University of Medicine and Pharmacy, Cluj-Napoca, Romania (no. 63/11 March 2019). A signed informed consent form was required before enrollment. The diagnosis of AIS was established by clinical and imaging criteria in accordance with the guidelines in place at that time [23].

The following inclusion criteria were set: patients diagnosed with new AIS confirmed by imaging techniques (head CT performed within one hour of admission to the emergency unit), who signed a written informed consent form.

Exclusion criteria were patients with hemorrhagic stroke, history of ischemic stroke, receiving vitamin K antagonists, active cancer, autoimmune diseases, liver cirrhosis, heart failure, history of acute myocardial infarction, acute coronary syndrome, and end-stage renal disease. Patients who died in the first 30 days after the AIS were also excluded.

Demographic, clinical, and laboratory data were noted for each patient: age, gender, living environment, smoking status, body mass index, presence of obesity, ischemic heart disease (IHD), arterial hypertension (AH), AF, DM, and dyslipidemia. Blood lipid profiles were also recorded. The National Institute of Health Stroke Scale (NIHSS) rating was calculated by an experienced neurologist. The presence of carotid plaques was also noted. The plaques characterized by the following were noted: protrusion of the carotid wall, thicker > 1.5 mm, or more than 50% of the intima-media thickness of the adjacent area [24]. The ischemic lesion volume was manually calculated by using the ellipsoid volume formula V = 4/3 π × (A/2) × (B/2) × (C/2), where A is the greatest diameter in the axial plane, B is the diameter at 90º to A in the axial plane, and C is the craniocaudal diameter. Measurements were performed by a radiologist and neurologist trained in neuroimaging. We selected the ellipsoid method because it provides a rapid, reproducible estimate of infarct volume on routine head CT without dedicated software. The approach is frequently used in AIS volumetry and is easy to standardize across raters.

Peripheral blood was collected in a vacutainer containing ethylenediaminetetraacetic acid. DNA extraction was performed using a genomic DNA purification kit (Wizard Genomic DNA Purification Kit; Promega, Madison, WI, USA) following the manufacturer’s instructions. Genotyping of the *VKORC1* rs9923231 (-1639G > A; VKORC1*2), *CYP4F2* rs2108622 (1347C > T), and *GGCX* rs11676382 (12970C > G) polymorphisms was performed as previously described.

Patient management was in accordance with the European guidelines at that time. Reperfusion therapy with alteplase was administered to eligible patients within 4.5 h of symptom onset after exclusion of intracranial hemorrhage on head CT. Aspirin (160–325 mg) was administered within 24 to 48 h. In patients with AF, direct oral anticoagulants were introduced. AH was treated with the appropriate drugs, taking into consideration the comorbidities. Statin therapy was prescribed unless contraindicated. Early mobilization and physiotherapy were initiated following the department protocol.

Mortality was noted if it occurred within five years following the 30 days after discharge, using the National Health Insurance House registry.

Statistical analysis was performed using MedCalc^®^ Statistical Software version 23.1.6 (MedCalc Software Ltd., Ostend, Belgium; https://www.medcalc.org; 2025). Nominal data were expressed as frequency and percentage. Normality of the distribution for quantitative data was assessed using the Shapiro–Wilk test. Non-normally distributed data were represented as median and 25–75 percentile. Comparisons between groups were performed using Mann–Whitney or chi-square tests, whenever appropriate. ROC analysis was used to establish a cutoff value for the association of several variables with mortality. Variables that achieved a *p*-value of <0.05 in the univariate analysis were introduced in the multivariate logistic regression. Multivariate logistic regression was used to identify variables that were independently associated with mortality. Statistical significance was considered at *p* < 0.05 in univariate analysis, and *p* < 0.007 for multivariate analysis (Bonferroni correction).

## 3. Results

We recorded 71 (28.1%) deaths after 5 years after enrollment. Table 1 shows the comparisons between survivors and deceased, regarding clinical, biochemical, and imagistic variables. Deceased patients tended to be older, much more often females, with lower triglycerides, an increased prevalence of carotid plaques, a larger lesion volume, were less likely to have received thrombolysis, had a higher NIHSS score, and were more likely to harbor the *VKORC1* homozygous genotype. Baseline comparisons in Table 1 are unadjusted and therefore descriptive. The *p*-values from this univariate analysis should not be interpreted as independent effects.

ROC analysis showed the cutoff values for mortality (Table 2). For age, a cutoff value of 72 years was calculated for 5-year mortality. For lesion volume, a cutoff of >12.63 mL was calculated for 5-year mortality. For NIHSS, a cutoff value of 7 was calculated for 5-year mortality. For TG a cutoff value of 110 mg/dl was calculated for 5-year mortality.

Multivariate logistic regression was employed to find out which variables are independently associated with mortality (Table 3). After applying the Bonferroni correction, advanced age > 72 years, volume > 12.63 mL, and a NIHSS score > 7 were the only variables independently associated with death. *VKORC1* (-1639G > A) SNP m/m variant was only marginally associated with mortality. Thrombolysis was also a marginally protective factor.

## 4. Discussion

In this study, we investigated the predictive role of several polymorphisms implicated in the VK metabolism (*VKORC1* rs9923231, *CYP4F2* rs2108622, and *GGCX* rs11676382) for 5-year mortality in patients with AIS. The results showed that the *CYP4F2* and *GGCX* variants are not associated with long-term mortality. The *VKORC1* polymorphism was only marginally associated with a higher risk of death. Advanced age, stroke severity, and lesion volume were the most important predictive factors for 5-year mortality in patients with AIS. To our knowledge, this is the first study to observe the potential predictive role of these polymorphisms for long-term mortality in patients not following a VK antagonists’ treatment.

VK helps maintain vessel homeostasis by activating protective proteins via γ-carboxylation: VKORC1 recycles VK, GGCX performed the activation step, and CYP4F2 influences VK breakdown. When this complex system does not function adequately, proteins like MGP and Gas6 will function improperly, which may favor vascular calcification and endothelial dysfunction and therefore could affect stroke recovery. The genes, whose polymorphisms were studied in our research (*VKORC1* rs9923231, *CYP4F2* rs2108622, and *GGCX* rs11676382), have specific roles in vitamin K homeostasis (hepatic and extra-hepatic), and their mutation can affect several VK-dependent proteins [25]. The *VKORC1* rs9923231 variant is responsible for a reduced regeneration of vitamin K hydroquinone from vitamin K epoxide [15]. This causes a decrease in the γ-glutamyl carboxylation of VK-dependent proteins by GGCX, including MGP, Gas6, protein S, and several coagulation factors (II, VII, IX, and X) [26]. The *GGCX* rs11676382 polymorphism reduces the catalytic efficiency even further, which can result in biologically inactive proteins due to their undercarboxylated nature (MGP and Gas6) [17]. MGP is one of the most potent endogenous inhibitors of arterial calcification. It binds calcium ions and prevents the accumulation of hydroxyapatite crystal in the vascular media [27]. If MGP is inactivated or its activity is reduced, it can lead to an accelerated vascular smooth muscle cell transformation into a specific osteoblast-like cell, an extracellular matrix remodeling, and increased arterial stiffness [28]. These pathways are strongly associated with recurrent ischemic events and higher cardiovascular mortality [29,30,31,32]. Gas6 is a secreted, vitamin K-dependent protein which binds itself to TAM (Tyro3, Axl, and MerTK) receptors, which are important for maintaining homeostasis, particularly of the immune, nervous, and vascular systems. Through TAM receptors, Gas6 is implicated in the mediation of several anti-apoptotic, anti-inflammatory, and endothelial-protective pathways [33]. An undercarboxylated Gas6 has decreased affinity for TAM receptors and it reduces survival signaling in vascular smooth muscle cells, endothelial cells, and neurons [34,35]. These disruptions contribute to endothelial barrier dysfunction, increased leukocyte adhesion, and compromised neurovascular repair in the post-ischemic phase and have been linked to an increased risk of recurrent vascular events and long-term mortality in AIS survivors [36,37,38,39]. The *CYP4F2* rs2108622 polymorphism reduces the hydroxylation of VK1, which can shift its distribution between liver and extrahepatic tissues [18]. This can impair the MGP and Gas6 activation even further. An altered VK metabolism is associated with upregulation of pro-inflammatory cytokines and oxidative stress, resulting in an accelerated atherosclerosis and increased risk of carotid plaque rupture [40,41]. During AIS, these chronic vascular changes may predispose patients to recurrent ischemic events, hemorrhagic transformation, and impaired functional recovery, all of which increase the risk of long-term mortality. In our cohort, carriers of the m/m *VKORC1* genotype were associated with a higher risk of long-term mortality. However, this association did not remain statistically significant after correction for multiple testing. This might be due to the limited sample size, as well as the modest effect size of this genetic variant when we introduced into multivariate analysis the stronger predictors such as age, lesion volume, or stroke severity. In a previous study we found the *VKORC1* polymorphism was only marginally associated with the risk of AIS [14]. The results of our previous study as well as this study suggest that the effect of *VKORC1* polymorphisms on AIS patients’ survival is indirect, mediated through vascular calcification or impaired activation of VK–dependent proteins. Larger, multi-center studies are needed to clarify if the observed trend represents a true biological effect.

A larger lesion volume was the most powerful predictor for long-term mortality in our study. Larger infarction areas are strongly associated with acute neurological deterioration, but also with a higher degree of neurological deficit, which may predispose AIS patients to several severe complications such as hemorrhagic transformation, extensive cerebral edema, and various infections [42,43]. The results complete our previous studies performed on different cohorts regarding the predictor role of lesion volume for in-hospital mortality of AIS patients and are in accordance with the literature findings regarding long-term mortality [44,45,46,47,48].

Advanced age is associated with severe AIS, larger infarction volumes, and it is accompanied by comorbidities like AH, AF, diabetes and/or chronic kidney disease. These factors highly reduce the survivability after an AIS. There are several arterial dysfunctions that are age-related and that might increase the recurrence of stroke and poor recovery: increased arterial stiffness and endothelial dysfunction [37,49]. Elderly patients have a decreased capacity for neuroplasticity and functional compensation after an AIS, which can contribute to disability and increased mortality rate [50]. The results of our study showed that advanced age was the second most important predictor for long-term mortality of patients after an AIS, and are consistent with several other studies [19,46,51].

The NIHSS score is a well-established and powerful predictor of long-term mortality in AIS patients, as it quantifies the stroke severity. A higher NIHSS score was independently associated with an increased 5-year mortality in AIS patients in our study. A high NIHSS score reflects greater neurological damage and is also a significant predictor for severe complications, like hemorrhagic transformation, cerebral edema, or infections, which increase the risk of short- and long-term mortality [19,44,52,53].

In our study, lower triglyceride levels were associated with higher mortality in univariate analyses, but not in multivariate regression. Even though high levels of triglycerides are considered a risk factor for stroke, there are several studies that show that low levels of triglycerides are linked to worse NIHSS, stroke complication, and increased mortality rate (the triglyceride paradox) [54,55,56]. Low triglycerides in AIS can be encountered in older patients, with a degree of sarcopenia, frailty and low energy reserves [57]. Those patients are more likely to develop infections, have poor wound healing, and, also, reduced potential for rehabilitation after AIS [58].

Gender was associated with mortality in our study only in univariate analysis. Women had a greater risk of 5 years mortality in our cohort. A meta-analysis showed that short and long-term mortality rates are significantly higher in women [59]. This likely due to older age at AIS, worse pre-stroke functional status, more severe index events, higher atrial fibrillation prevalence, and a higher prevalence of post-stroke depression [60].

BMI was lower in patients that died than in survivors, but the differences was only close to statistical significance. It likely reflects the frailty, sarcopenia, and low energy reserves. This data is consistent with studies that showed that lower BMI is associated with worse post-stroke disability and higher mortality [61].

The study has several strengths. It is, to our knowledge, the first study to evaluate the potential predictive role of VKORC1, CYP4F2, and GGCX polymorphisms for 5-year mortality in AIS patients who were not treated with VK antagonists. The patients were followed for a relatively long period, and the analysis included genetic, clinical, demographic, and imaging variables. Several limitations must be acknowledged. The sample size was relatively modest due to the extensive exclusion criteria. The single-center design may reduce the generalizability of our findings to other populations with different genetic or environmental backgrounds. At that time, few patients benefited from thrombolysis, but although the procedure is applied more frequently at present, there are still many cases that do not benefit from the procedure. Another limitation is the absence of systematically collected pre-stroke modified Rankin Scale. Larger multicenter studies are needed to validate our results.

Our results could have several practical implications. Common clinical and imaging data, such as age, NIHSS, and lesion volume, should be considered in any predictor tools for stratifying the risk of 5-year mortality. Genetic markers, like polymorphisms involved in VK metabolism, should not be determined routinely. Their practical value, especially VKORC1, will be determined in larger, multicenter studies. Future work should investigate the prognostic value of biochemical markers that quantify disturbances in VK metabolism (e.g., MGP, PIVKA-II).

## 5. Conclusions

In this study, we included AIS patients and showed that *VKORC1* rs9923231, *CYP4F2* rs2108622, and *GGCX* rs11676382 did not independently predict mortality. The *VKORC1* variant was only marginally associated with mortality, but this was attenuated after correction for multiple testing. Advanced age, NIHSS score, and the lesion volume were independent predictors of long-term mortality of AIS patients. These results suggest that these polymorphisms implicated in VK metabolism, although biologically plausible, add limited prognostic value in addition to the established clinical and imaging variables. Larger multicenter studies are needed to validate the results.

## Figures and Tables

**Table 1:** Comparison of survivors and deceased patients.

Variables		Survivor Group (N = 181)	Deceased Group (N = 71)	*p*-Value
Age (median, IQR)		70 (61; 79)	78 (73; 85)	<0.001
Gender (N, %)	Male	81 (44.8)	12 (16.9)	<0.001
	Female	100 (55.2)	59 (83.1)	
Environment (N, %)	Urban	114 (63)	42 (59.2)	0.6
	Rural	67 (37)	29 (40.8)	
Smoking (N, %)		51 (28.2)	13 (18.3)	0.1
Obesity (N, %)		40 (22.1)	13 (18.3)	0.6
BMI (median, IQR)		26.9 (24.3; 29.4)	25.8 (23.8; 29)	0.08
AH (N, %)		152 (84)	61 (85.9)	0.8
AF (N,%)		26 (14.4)	16 (23.2)	0.1
IHD (N, %)		25 (13.8)	7 (9.9)	0.5
DM (N, %)		40 (22.1)	17 (23.9)	0.8
Dyslipidemia (N, %)		164 (90.6)	68 (95.8)	0.1
TC (mg/dL) (median, IQR)		183 (155; 207)	168 (132; 210)	0.1
HDL-C (mg/dL) (median, IQR)		44 (38; 54)	45 (37; 54)	0.7
LDL-C (mg/dL) (median, IQR)		105 (80.7; 129.6)	101.6 (74.6; 131.6)	0.7
TG (mg/dL) (median, IQR)		123 (93.5; 170)	95 (73; 123)	<0.001
Carotid plaque (N, %)		100 (55.2)	56 (78.9)	0.001
Lesion volume (mL)		10.5 (5.8; 25.44)	18.8 (10.8; 34.2)	<0.001
Thrombolysis (N, %)		32 (17.7)	5 (7)	0.03
NIHSS		5 (3; 7.5)	8 (5; 14.2)	<0.001
*CYP4F2* (1347C > T) SNP (N, %)	w/w	80 (44.2)	30 (42.3)	0.9
	w/m	80 (44.2)	33 (46.5)	
	m/m	21 (11.6)	8 (11.3)	
*GGCX* (12970C > G) SNP (N, %)	w/w	162 (89.5)	62 (87.3)	0.7
	w/m	19 (10.5)	9 (12.7)	
*VKORC1* (-1639G > A) SNP (N, %)	w/w	53 (29.3)	12 (16.9)	0.01
	w/m	105 (58)	40 (56.3)	
	m/m	23 (12.7)	19 (26.8)	
*VKORC1* (-1639G > A) SNP (N, %)	w/w or w/m	158 (87.3)	52 (73.2)	0.01
	m/m	23 (12.7)	19 (26.8)	

N: number; IQR: interquartile range; BMI: body mass index; AH: arterial hypertension; AF: atrial fibrillation; IHD: ischemic heart disease; DM: diabetes mellitus; TC: total cholesterol; HDL-C: high-density lipoprotein cholesterol; LDL-C: low-density lipoprotein cholesterol; TG: triglycerides; w: wild-type; m: mutated; SNP: single nucleotide polymorphism.

**Table 2:** ROC analysis of predictors of mortality.

Variable	Cutoff	AUC (95CI)	Se	Sp	*p*
Age	>72 years	0.706 (0.645–0.761)	78.8 (67.6–87.7)	57.4 (49.9–64.8)	<0.001
Lesion volume	>12.63 mL	0.646 (0.583–0.705)	67.6 (55.5–78.2)	61.8 (54.4–69.0)	<0.001
NIHSS	>7	0.702 (0.641–0.757)	54.2 (41.9–66.3)	75.1 (68.2–81.3)	<0.001
TG	<110 mg/dL	0.676 (0.615–0.734)	69 (56.9–79.5)	60.7 (53.3–67.9)	<0.001

AUC—area under the curve, Se—sensitivity, Sp—specificity, TG: triglycerides.

**Table 3:** Multivariate logistic regression for mortality.

Variable	B	*p*	OR	95% C.I. for OR	
				Min	Max
Age > 72 years	1.04	0.007	2.83	1.32	6.08
Gender (male)	−0.76	0.08	0.46	0.19	1.10
Lesion volume > 12.63 mL	1.40	<0.001	4.05	2.05	7.99
*VKORC1* (-1639G > A) SNP m/m	0.88	0.03	2.41	1.04	5.59
NIHSS score > 7	0.97	0.006	2.64	1.31	5.31
Carotid plaque	0.47	0.26	1.61	0.69	3.72
Thrombolysis	−1.21	0.04	0.29	0.09	0.95
TG < 110 (mg/dL)	0.63	0.06	1.88	0.95	3.74
Constant	−1.38	<0.001	0.25		

B—regression coefficient (log-odds); OR—odds ratio; CI—confidence interval; TG—triglycerides.

## Data Availability

The datasets presented in this article are available upon reasonable request from the corresponding author.
